# Novel biomarkers for prognosticating diabetic kidney disease progression

**DOI:** 10.1007/s11255-022-03354-7

**Published:** 2022-10-22

**Authors:** Shilna Muttickal Swaminathan, Indu Ramachandra Rao, Srinivas Vinayak Shenoy, Attur Ravindra Prabhu, Pooja Basthi Mohan, Dharshan Rangaswamy, Mohan V Bhojaraja, Shivashankara Kaniyoor Nagri, Shankar Prasad Nagaraju

**Affiliations:** 1 Department of Nephrology, Kasturba Medical College, Manipal, Manipal Academy of Higher Education, Manipal, Manipal, India; 2 Department of Gastroenterology, Kasturba Medical College, Manipal, Manipal Academy of Higher Education, Manipal, Manipal, India; 3 Department of Medicine, Kasturba Medical College, Manipal, Manipal Academy of Higher Education, Manipal, Manipal, India

**Keywords:** Diabetic kidney disease, Albuminuria, Novel biomarkers, eGFR

## Abstract

The global burden of diabetic kidney disease (DKD) is escalating, and it remains as a predominant cause of the end-stage renal disease (ESRD). DKD is associated with increased cardiovascular disease and morbidity in all types of diabetes. Prediction of progression with albuminuria and eGFR is challenging in DKD, especially in non-proteinuric DKD patients. The pathogenesis of DKD is multifactorial characterized by injury to all components of the nephron, whereas albuminuria is an indicator of only glomerular injury. The limits in the diagnostic and prognostic value of urine albumin demonstrate the need for alternative and clinically significant early biomarkers, allowing more targeted and effective diabetic treatment, to reduce the burden of DKD and ESRD. Identification of biomarkers, based on multifactorial pathogenesis of DKD can be the crucial paradigm in the treatment algorithm of DKD patients. This review focuses on the potential biomarkers linked to DKD pathogenesis, particularly with the hope of broadening the diagnostic window to identify patients with different stages of DKD progression.

## Introduction

Diabetic kidney disease is a common cause of end-stage renal disease (ESRD), which occurs in 20–40% of all diabetics [1]. The global burden of disease study estimated that the all-age mortality from CKD raised to 41.5%, with diabetes being one of the major risk factors, contributing to half of the death from CKD [2]. This increased death rate associated with DKD has been linked to micro-and macroangiopathies. The alarming rise in DKD prevalence demands timely detection of disease progression, allowing for more targeted and effective treatment.

The clinical diagnosis of DKD was conventionally based on significant albuminuria, in type -1 or type-2 diabetes patients, but this practice is questionable as these patients have non-proteinuric kidney disease, in which the kidney malfunction does not remarkably lead to the presence of albuminuria in DKD patients [3]. This non-classic phenotype accounts for a prevalence of 20% to 40% of all DKD, suggesting that proteinuria does not always occur preceding the loss of renal function in diabetes [4].

DKD is a progressive disease with multifactorial pathogenesis involving glomerular, tubular, and inflammatory changes resulting in irreversible renal fibrosis [5]. Hyperglycemia, smoking, dyslipidemia, hypertension, and obesity are the major risk factors associated with DKD [6]. The spectrum of DKD involves nearly every nephron structure: glomerular endothelia and epithelia, podocytes, mesangial matrix, as well as renal tubular epithelia [7]. Thus, it is time for the identification of biomarkers beyond proteinuria, based on the pathophysiology of DKD can be the crucial paradigm in the treatment algorithm of DKD patients. Over the last few years, remarkable progress has been made in comprehending the pathophysiology of DKD, along with the explication of related biomarkers. So far, several promising novel biomarkers linked to the pathogenesis site have been emerged from clinical studies. Many of these biomarkers potentially diagnose DKD early, and few are significant in predicting renal function decline. This review will reiterate the clinical relevance of multiple potential biomarkers involved in the pathogenesis of DKD progressions, such as glomerular injury, tubular injury, oxidative stress, and inflammation.

## Pathogenesis of DKD (Fig. 1)

**Fig. 1:**
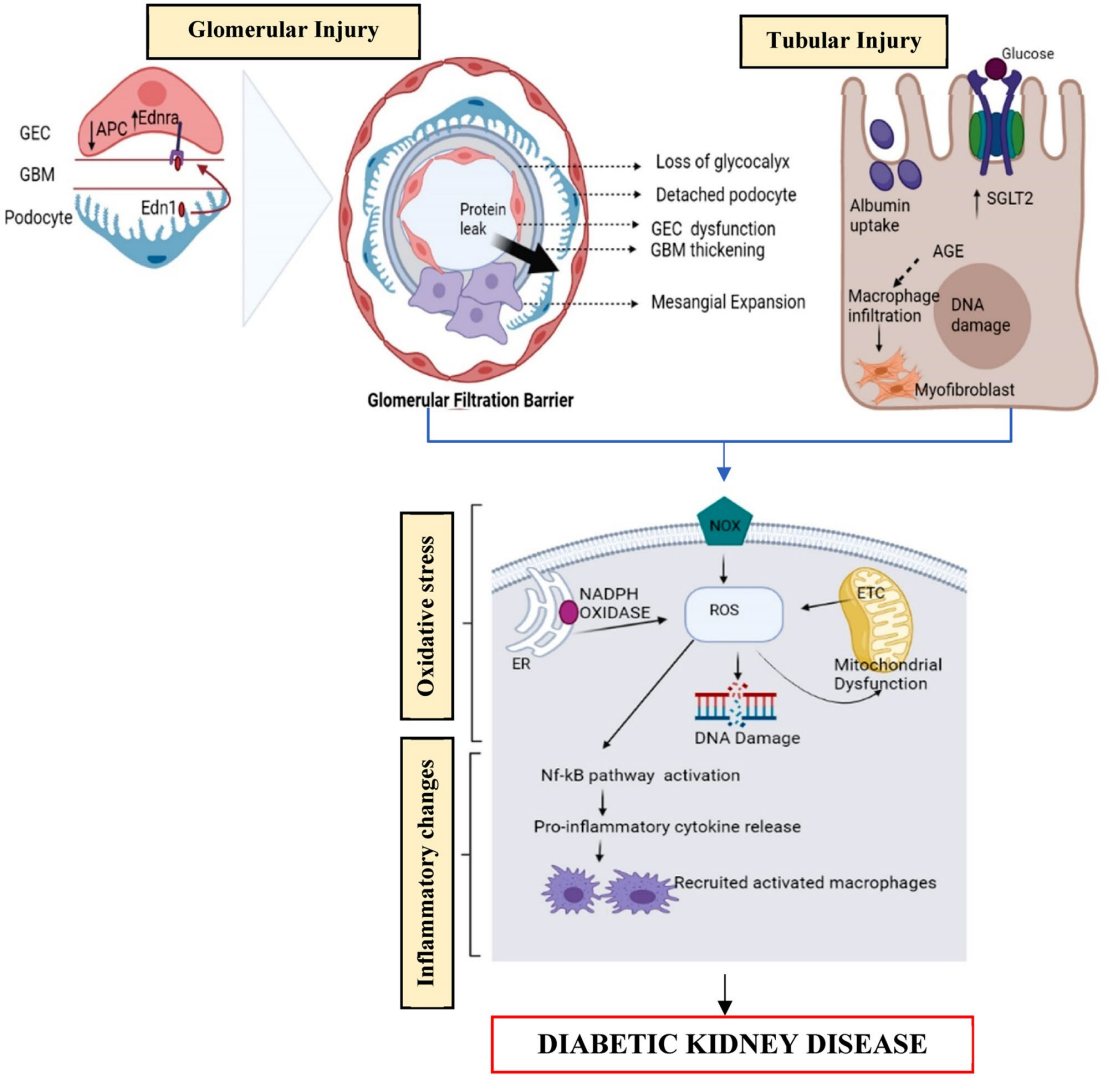
Overview of diabetic kidney disease pathophysiology. Diabetic milieu alters glomerular permeability by reducing the production of activated protein kinase C and activation of the endothelin-1 and endothelin-1 receptor leading to glomerulus morphological changes and AGE production. In tubular epithelial cells, upregulated SGLT2 and increased albumin reabsorption culminate into cell toxicity and further AGE production leading to activation of pro-inflammatory cytokines and tubulointerstitial fibrosis*. GEC* glomerular endothelial cell, *GBM* glomerular basement membrane, *APC* activated protein C, *Edn1* endothelin-1, *Ednra* endothelin-1 receptor A, *SGLT2* sodium-glucose transport protein 2, *AGE* advanced glycation end-product, *NOX* nicotinamide adenine dinucleotide phosphate oxidase

The cellular heterogeneity and the different physiological functions of the kidney make DKD progression a complicated process. Chronic hyperglycaemia activates several pathological processes that affect glomerular endothelial cells, smooth muscle cells, mesangial cells, and podocytes. DKD has multiple stages of development, and widely recognized mechanisms are hyperglycemia-induced metabolic and hemodynamic pathways. Hemodynamic changes increase systemic and intra-glomerular pressure, thereby stimulating vasoactive hormone pathways, while metabolic changes contribute to mesangial cell expansion, mesangial cell apoptosis, and structural changes. These pathways converge ultimately, leading to inflammation, endothelial dysfunction, and fibrosis [8].

### Glomerular injury

The glomerulus is the principal location of diabetic kidney injury. Diabetic milieu reduces the production of activated protein C via suppression of thrombomodulin expression, affecting the glomerular permeability and enhancing the apoptosis of glomerular endothelial cells and podocytes. On activation of the endothelin-1 and endothelin -1 receptor, depletion of endothelial nitric oxide and the destruction of the glycocalyx occurs. The signs of progressive DKD include changes such as glomerular basement membrane thickening, podocyte foot process effacement, and intra-glomerular mesangial cell expansion resulting in the reduction of the glomerular surface area [9].

### Tubular injury

Sodium-glucose transport protein 2(SGLT2) in proximal tubules facilitates more than two-thirds of sodium and glucose reabsorption under normal conditions. Hyperglycemia induces upregulation of SGLT2, leading to increased glucose reabsorption, which predisposes proximal epithelial cells to a hypoxic injury and causes advanced glycation end-product (AGE) production. Similarly, increased albumin reabsorption by proximal tubules culminates into proximal epithelial cell toxicity [10]. Thus, high diabetic-milieu and AGE facilitate pro-inflammatory and apoptosis cascade leading to tubulointerstitial injury and fibrosis.

### Oxidative stress injury

Reactive oxygen species (ROS) and reactive nitrogen species (RNS) are responsible for oxidative stress, leading to disease progression. Mitochondria and the nicotinamide adenine dinucleotide phosphate oxidase (NOX) family are the primary sources of reactive oxygen stress in the kidney. It primarily gets produced by enzymatic reactions, from mitochondrial aerobic respiration (ETC) and a lesser amount from endoplasmic reticulum and peroxisomes [11].

Oxidative stress is also contributed from hyperglycemia-induced AGEs production in the later stages of non-enzymatic glycation of sugar and protein, by the process called Maillard reaction [11]. Hyperglycemia-induced oxidative stress mediates DNA damage, lipid peroxidation, mitochondrial dysfunction, and infiltration of inflammatory cells, progressing to renal cell damage [8].

### Inflammatory injury

DKD progression is accelerated by the activation of the nuclear factor-kappa light chain enhancer for B cells (Nf-kB) by an activated immune system and inflammation. Macrophages, dendritic cells, and mast cells make up the renal mononuclear phagocytic cells (MNPs) which consist of macrophages, dendritic cells, and mast cells are involved from the innate immune system. Macrophage infiltrations are prominent in glomeruli and interstitium of DKD patients [12]. Its accumulation in glomeruli leads to glomerulosclerosis and in interstitium predicts loss of GFR. Glomerular infiltration of dendritic cells is proportional to proteinuria during the progression of DKD. While mast cell infiltration of the interstitium is correlated with serum creatinine, but not with proteinuria. These mast cell degranulation and IL-1β from macrophages together stimulate renal fibroblast proliferation, whereas IL-1 stimulates mesangial cell proliferation [12].

The cellular arm of adaptive immunity involved in DKD is T cells (Th1, Th17) and limited involvement of B cells, which are associated with proteinuria [12].

## Existing biomarkers

Albuminuria and eGFR are the commonly used legacy markers of renal function decline in routine clinical practice, although they lack specificity and sensitivity in predicting the DKD progression in diabetic patients. Proteinuria does not always precede renal function decline, suggesting early involvement of tubulointerstitial compartment rather glomerular [4, 13]. On the other hand, eGFR estimation also has some downsides as a biomarker in diagnosing and stratifying DKD progression, since its calculation using serum creatinine interferes with the patient's muscle mass and meat diet. eGFR estimation using the CKD-EPI equation also gives underestimated values in type 2 diabetic patients [14]. Understanding these limitations and exploring potential biomarkers is necessary for both clinical applications in future research for improved diagnostic and prognostic tools.

## Novel biomarkers in DKD

Over the past decades, immense efforts in research have been carried out to validate alternative biomarkers. Numerous biomarkers were identified for this purpose, and initial findings from many studies have been promising. These novel biomarkers can be classified according to the pathological effects on renal structure, as shown in Table 1.

**Table 1:** Classification of biomarkers

Glomerular biomarkers	Tubular biomarkers	Biomarkers of oxidative stress	Biomarkers of inflammation
Type IV collagen	NGAL	8oxodG	Tumor necrotic factor-**α**
Fibronectin	Urinary cystatin C	Pentosidine	Tumor necrotic factor-α receptors
Laminin	KIM-1	Uric acid	MCP-1
Serum cystatin C	RBP4		TGF- β
Glycosaminoglycans	L-FABP		CTGF
Immunoglobulin G			IL-6
Ceruloplasmin			
L-PGDS			
Transferrin			

*L-PGDS* lipocalin-type prostaglandin D synthase, *NGAL* Neutrophil gelatinase-associated lipocalin, *KIM-1* kidney injury molecule-1, *RBP4* retinol-binding protein. *L-FABP* liver-type fatty acid-binding protein, *8oxodG* 8-Oxo-7,8-dihydro-2-deoxyguanosine, *MCP-1* monocyte chemoattractant protein, *TGF-β* transforming growth factor-Beta, *CTGF* connective tissue growth factor, *IL-6* Interleukins-6

## Glomerular biomarkers

Biomarkers linked to glomerular injury would be a significant tool in guiding early diagnosis and identifying patients with rapid renal deterioration. Multiple glomerular biomarkers provide great evidence in representing glomerular injury as urine protein estimation alone cannot predict the progression of DKD.

### Type IV collagen

Structurally Type 1 V Collagen is a protein with three polypeptide α-chains in triple helix form which serves as the main basement membrane constituent of the glomerulus, tubules, and mesangial matrix [15]

Mesangial expansion score and tubulointerstitial injury score were statistically correlated with urinary type IV collagen, suggesting the pathogenic processes of DKD reflected in the elevation of this protein [15]. Tomino et al., in an Asian multicentre study, observed a gradual rise in urinary Type IV collagen from normo-micro-macroalbuminuric stages as the disease progressed [16]. Ijima et al. studied the urinary type IV collagen in the normo-microalbuminuric group, excluding overt proteinuria, and after 1-year follow-up, the normoalbuminuric group with a higher level of urinary type IV collagen excretion had developed microalbuminuria [17]. The findings of the above studies suggest the importance of type IV collagen as a biomarker in diagnosing the onset of microalbuminuria. Additionally, Morita et al. argue with their findings among the T1DM population that type IV collagen was independently associated with microalbuminuria [18]. However, Araki S et al., in a follow-up study with T2DM patients, did not exhibit a significant change of type IV collagen with the progression of DKD [19]. While, serum type IV collagen was found higher in diabetic retinopathy, indicating its involvement in predicting microvascular complications [20].

From the above-mentioned studies, urinary type 1V collagen is an indicator of early onset and disease progression in both type-1 and type-2 diabetic patients, and its higher concentration in the serum indicates the onset of diabetic nephropathy.

### Fibronectin (FN)

Fibronectin is a fibrillar protein on the cell surface, and its soluble form in plasma is associated with constriction of the glomerular extracellular matrix. It is primarily synthesized in fibroblast and endothelial cells, and its upregulation in capillary and mesangium of the glomerulus in diabetic patients has been reported [21].

Plasma fibronectin was found progressively increasing from normoalbuminuric to microalbuminuric patients [21]. Marked elevation of urinary fibronectin (U-FN) was associated with overt proteinuria in both T1DM and T2DM [22, 23]. Urinary and plasma fibronectin were found to be linked with micro- and macrovascular complications such as retinopathy, neuropathy, and cardiovascular incidence among diabetic patients [22, 24]. All these studies demonstrated predictive performance of FN for microalbuminuria and overt proteinuria, in addition to micro-macrovascular complications in type 1 and type 2 diabetic patients.

Although a great number of researchers found increased U-FN in DKD, the exact origin of FN remains unclear, as it was synthesized from multiple sources other than renal cells. Further clinical research is necessary to compare it to albuminuria and to determine its significance.

### Laminin

Laminin is an adhesive and non-collagenous component of glomerular basement membranes and mesangium. To date, 15 laminin isoforms are identified in which A2 laminin reflects mesangial matrix expansion in DKD [25].

Banu et al. have demonstrated a higher level of laminin in normoalbuminuric patients, suggesting that it would be a marker for predicting albuminuria, and found a positive correlation with tubular dysfunction markers including NAG and **α-**1microglobulin [26]. Rashed et al. have observed progressively increasing serum laminin with 135.7 pg/ml cut-off value in type 2 diabetic patients, in ROC analysis to demonstrate the clinical diagnostic utility [27] Furthermore, a significant rise in serum concentration of laminin has been reported with worsening diabetic retinopathy as it is secreted from endothelial cells [28].

In aggregate, serum laminin would be a marker for identifying the onset and progression of diabetic kidney disease as well as diabetic microangiopathy.

### Cystatin C (CysC)

CysC is a low-molecular-weight protein that acts as an endogenous cysteine proteinase and is identified as a potential surrogate indicator for GFR estimation, because, unlike serum creatinine, it does not influence extrarenal factors [29] which leads to the increased diagnostic utility of serum CysC to evaluate kidney damage, reflecting directly to GFR.

Diagnostic utility of serum CysC in normoalbuminuric patients has also been well documented in a cohort of T1DM, indicating the predictive performance of CysC before the renal dysfunction appears [30] which is well supported by Qamar et al. in type 2 diabetic patients with a sensitivity of 88.2% and specificity of 84.8% [31]. In multiple studies done among CKD patients with T1DM and T2DM, showed a significant role of serum CysC as a predictor of progression to ESRD [32]. Clinical research has observed the positive association of serum CysC with retinopathy [33] and cardiovascular risk [34] in type 2 DM. Nevertheless, as recommended in current KDIGO guidelines, CysC would result in increased health care costs [35].

The above studies displayed that serum cystatin C could be a promising biomarker for early diagnosis and for predicting the progression of DKD, as it is a strong predictor of microvascular and macrovascular complications of diabetes.

### Glycosaminoglycans (GAG)

GAGs are mucopolysaccharides (13 and 30 kDa). The most prevalent types are chondroitin and dermatan sulfate, keratan sulfate, heparan sulfate, and heparin. Heparan sulfate gives negative potential to the glomerular basement membrane (GBM) to control the perm selectivity of the glomerulus. In DKD, endothelial dysfunction leads to loss of these functional groups which gives rise to hyperfiltration resulting in albuminuria [36]. The Steno hypothesis postulates a defect in heparan sulfate regulation in the glomerulus, which determines the high susceptibility of diabetic patients to develop proteinuria and eventually induces the excretion of heparan sulfate in the urine [37]. Many experimental studies supporting this hypothesis observed a rise in urinary GAG excretion, especially heparan sulfate in T1DM and T2DM patients with microalbuminuria and macroalbuminuria than the normoalbuminuric group, suggesting that GAG is a vital screening predictor of microalbuminuria [38].

The functional role of GAG in permeability properties of retinal basement membrane has also been documented. Kahaly et al. have evaluated high urinary GAG concentration in diabetic nephropathy patients with retinopathy complications [39]. Linked with these findings, Budak et al. have also reported a positive correlation of GAG with diabetic retinopathy [40].

Urinary GAG evaluation could be a vital marker for predicting the onset of microalbuminuria in type 1 and type 2 diabetic patients and microvascular complications of diabetes in the progressive stages.

### Immunoglobulin G (IgG)

IgG is a 150 kDa anionic immunoprotein in serum [41]. The urinary excretion of this high-molecular-weight protein indicates increased GBM porosity with large shunt-like pores and podocyte deficiency and effacement of foot processes. Thus, IgG appears in progressive DKD when severe irreversible kidney lesions occur [41].

Yashima et al. have reported a significant elevation of urinary IgG in normoalbuminuric DKD patients among the T2DM population and also observed a correlation with progressive diffuse glomerular lesions [42]. Multiple studies in type 2 diabetic patients have suggested that urinary level of IgG predicts the onset of microalbuminuria [41, 43]. Apart from using total IgG level, subtype levels and their ratio has been used as a marker of glomerular charge selectivity impairment. A cross-sectional study evaluated the level of IgG subtypes such as IgG_2_ and IgG_4_ ratio between T1DM and T2DM, which observed a higher level of IgG_2_/IgG_4_ in the latter [44].

Altogether, these studies suggest that measurement of urinary IgG could be an effective marker for predicting the onset of microalbuminuria in T2DM.

### Ceruloplasmin

Ceruloplasmin is a copper carrier and acts as a pro-oxidant in severe oxidant stress conditions. It has been studied as an independent risk factor for the incidence of cardiovascular diseases and insulin resistance [45]. Jung Lee et al. have found T2DM with a higher incidence of serum ceruloplasmin level in progressors than those in non-progressors, indicating an independent factor for progression [45].

Furthermore, urinary ceruloplasmin was also found elevated in T2DM before albuminuria appears [43]. A similar observation was found in T1DM [46]. This evidence suggests parallel evaluation of both serum and urine ceruloplasmin would be an independent predictive marker in DKD progression in T2DM. However, further studies require in the type 1 population, which is limited so far.

### Lipocalin-type prostaglandin D synthase (L-PGDS)

L-PGDS is a lipocalin secretory protein that synthesizes prostaglandin D2. It is primarily produced in the choroid plexus in the brain and discharged readily to circulating blood with chemical features like albumin, such as anionic charge, and can move quickly through the glomerular capillary due to its small molecular weight (20–31 kDa). Thus, urine L-PGDS reflect minor changes in the permeability of glomerular capillary walls [47].

Researchers have studied its utility in predicting the early stages of DKD by observing the elevated urinary L-PGDS in diabetic patients with normoalbuminuria [48]. There was a significant increase in urine L-PGDS than serum level in normo- or macroalbuminuria in parallelly in advanced DKD [49]. The findings of these studies suggest that evaluation of L-PGDS in urine could identify the early onset of DKD.

### Transferrin

Transferrin is a glycoprotein with two iron-binding domains, which is primarily produced in the liver. It is involved in multiple functions like iron transportation and immune regulation against micro-organisms [50]. Gonzalez et al. [51], reported that transferrin gets accumulated in the cytoplasm of glomerular podocytes in the early stages of DKD. Iron liberated from transferrin contributes to oxidative stress and insulin resistance in T2DM patients, and thus, dysregulation of iron homeostasis is associated with the development of DKD [50]. M. Kanauchi et al. in their study observed the correlation of urinary transferrin with progressive changes such as interstitial fibrosis, atrophic renal tubular cells, and infiltration of renal interstitium with inflammatory cells [52]. The renal accumulation of iron and excretion of transferrin in urine leads to a lower serum level, which indicates renal cell toxicity resulting from accumulated free iron [50].

Excretion of transferrin in urine has been reported in normoalbuminuric T2DM, indicating prediction at an early stage than albuminuria in DKD [43]. High excretion of urinary transferrin in normoalbuminuric and microalbuminuric patients among T1DM was reported by previous researchers [53]. These findings were supported by a retrospective cohort study which suggests that low serum transferrin was associated with ESRD in diabetic patients [50]. Additionally, studies have also been reported transferrinuria in predicting micro- and macrovascular complications of diabetes which leads to retinopathy [54] and cardiovascular disease [55].

These findings suggest that the increased urinary and lower serum level of transferrin in all diabetic patients would predict the development of microalbuminuria and significant indicator for complications of diabetes.

## Tubular markers

The renal tubules and interstitial compartments play a significant role in the development of DKD [56]. The extent of tubulointerstitial damage may determine renal function decline in diabetes, even in normoalbuminuric renal insufficiency [56]. Hence, the tubular indicators of kidney injury have a pivotal role to measure the degree of long-term kidney impairment in DKD patients.

### Neutrophil gelatinase-associated lipocalin (NGAL)

NGAL is a neutrophil granular constituent belonging to the lipocalin protein family. In renal injury, the distal tubules and collecting duct signify the higher expression of NGAL. It has been validated as an acute kidney injury (AKI) biomarker extensively. NGAL is involved in antimicrobial defense mechanisms and anti-apoptosis. It is a definite marker of acute renal damage, because a burnt-out nephron does not generate NGAL [57].

Evidences have been postulated on the significant role of NGAL in CKD and later in DKD [58] which has been supported by the findings of S. Hwang et al., where they have observed histological correlation of NGAL with progressive renal lesion [56].

Urinary NGAL (uNGAL)-to-creatinine ratio was found useful to differentiate DKD from non-diabetic kidney disease with high specificity (90.5%) [59]. Kaul et al. observed that the significant rise of serum NGAL (sNGAL) and uNGAL from normo-micro-macroalbuminuria in T2DM [57]. Peng He et al., in their meta-analysis, reported that sNGAL and uNGAL are significant biomarkers in the diagnosis of DKD [60]. Growing evidences have depicted a positive correlation of NGAL with albuminuria and other tubular markers, including RBP4, Cystatin C, and KIM-1, whereas it is negatively correlated with eGFR, suggesting the involvement of NGAL in DKD progression [61–63]. In contrast, Kim et al. reported that no significant difference in NGAL was found in normoalbuminuric and macroalbuminuric patients [64].

In summary, these clinical studies suggest that sNGAL and uNGAL could be valuable markers for diagnosing the onset of DKD and stratifying the disease into different stages. However, large-scale prospective studies are necessary to implement NGAL as a biomarker into routine clinical use.

### Urinary cystatin C (uCysC)

Cystatin C is a 13.4-kDa cysteine protease inhibitor generated by all nucleated cells. Normally, it is not present in urine significantly. Reduced reabsorption from injured/dysfunctional tubules causes fluctuations in urinary CysC levels [65]

Previous studies have demonstrated uCysC as a promising biomarker of tubular dysfunction in AKI [65], and it has been extensively studied in DKD [66]. Xian et al. have evaluated the diagnostic performance of uCysC in DKD and the onset of microalbuminuria among T2DM. With respect to DKD diagnosis, the area under the ROC curve was 0.803, and in diagnosing the onset of microalbuminuria, AUC was 0.805 with progressive elevation in uCysC from normo-micro-macroalbuminuria [61].

These studies suggest uCysC as a sensitive biomarker mirroring tubular impairment, which can be determined before the onset of microalbuminuria.

### Kidney injury molecule-1 (KIM-1)

In response to injury, KIM-I is predominantly expressed in the apical membrane of proximal tubular cells. Palmitic acid bounded albumin uptake by proximal tubules is being enhanced by KIM-1, leading to further tubulointerstitial damage [67]. Van Timmeren et al. demonstrated that KIM-1 mainly expresses the luminal side of dedifferentiated proximal tubular areas, which had more fibrosis and inflammation [67].

Several studies have documented an estimation of KIM-1 in urine (uKIM-1) as a predictive indicator for AKI as it appears well before serum creatinine increases [68]. Fourth, Ali et al. reported uKIM-1 with more specificity and sensitivity than urine albumin in diagnosing early stages of DKD [69], and Gohda et al. reported a significant association of serum KIM-1 with a lower GFR rate. [70] Clinical studies have highlighted that uKIM-1 values were gradually increased in patients with T1DM and T2DM from normo- micro-macroalbuminuria [70, 71].

Based on these studies, uKIM-1 appears to be a promising marker for diagnosing early onset and predicting the different stages of disease progression in type 1 and type 2 diabetic patients. Upon this, serum level estimation might be a sensitive marker for progression as GFR declines.

### Retinol-binding protein-4 (RBP4)

RBP4 is a low-molecular-weight protein associated with the lipocalin family, predominantly synthesized in the liver and adipose tissue. The main function of RBP-4 is to transfer small hydrophobic molecules to the cell membrane. In diabetes, an association of RBP4 concentration has been documented with the magnitude of insulin resistance, suggesting increased levels of RBP4 predicts insulin resistance [72].

Increased plasma and urinary RBP4 concentration have been reported with low eGFR [72] [73]. A longitudinal study among T1DM has reported an increased level of urinary RBP4 in microalbuminuric patients [74] and the diagnostic utility of urinary RBP4 in T2DM patients with an AUC of 0.74 has been established in another research [62]. The serum level of RBP-4 was found to be associated with proliferative diabetic retinopathy and coronary cerebrovascular or peripheral vascular diseases among type 2 diabetes [72, 75]. Discordant results were shown by E Akbay's study, indicating that diabetic retinopathy and cardiovascular complications do not exhibit any change in serum RBP4 in T2DM patients [76].

RBP4 could be a valid marker for identifying the early onset of DKD and predicting renal function impairment in progressive stages in T1DM and T2DM. In addition, this marker could be a predictor for microvascular and macrovascular complications of diabetes.

### Liver-type fatty acid-binding protein (L-FABP)

L-FABP is a 14 kDa protein produced mainly in the cytoplasm of proximal tubules and is involved in the metabolism of the long-chain fatty acids. Uncontrolled reabsorption of free fatty acids to tubular cells by L-FABP leads to tubulointerstitial damage [77]. According to Kamijo et al., after a 4-year follow-up of T2DM, urine L-FABP levels were found to be increasing progressively from normo-micro-macroalbuminuria and, further, increased in patients with ESRD. Higher levels of L-FABP in the normoalbuminuric group suggest that it could be a risk factor for disease progression [78]. Corroborating these findings, a 12-year follow-up study by Araki S et al. observed a significant elevation of the urinary L-FABP in T2DM who had 50% of GFR decline and incidence of cardiovascular disease [79]. Additionally, the urinary level of L-FABP offers statistical significance with urine albumin level and inversely correlates with GFR [78]. Thus, evaluation of urinary L-FABP in T2 DM serves as a risk factor for DKD progression and could be considered as a promising tubular marker in predicting the incidence of cardiovascular disease and renal function impairment.

## Biomarker of oxidative stress

Evidence from epidemiological and mechanistic research suggests that oxidative stress plays a key role in mediating progression and complications. Thereby, markers linked to ROS production have considerable potential to stratify DKD stages.

### 8-Oxo-7,8-dihydro-2’-deoxyguanosine(8-oxodG)

8-oxodG is an oxidized nucleoside of DNA produced due to oxidative stress in living cells. Numerous evidence has indicated urinary 8-oxodG is a risk factor for cancer, atherosclerosis, and diabetes [80]. Xu et al., in a study among T2DM patients with diabetic nephropathy, have found a higher level of urinary 8-oxodG in those who had microalbuminuria [81]. Clinical research with 5 years of follow-up reported significant progression of diabetic kidney disease in patients with higher urinary 8-oxodG [82]. Urinary 8-oxodG has been proposed as a characteristic pathogenic component in diabetic retinopathy development in T1DM and T2DM [83, 84]. Etiane et al. found diagnostic ability of 8-oxodG with an AUC of 0.836 to evaluate microvascular complications in diabetic patients [85]. This marker has also been observed with macrovascular complications in T2DM [86]. These above-mentioned findings conclude that excretion of urinary 8-oxodG could be an independent predictor for disease progression and development of microvascular and macrovascular complications of diabetes.

### Pentosidine

Pentosidine is an advanced glycoxidation product formed by the covalent binding of amino groups with glucose moiety [87]. Miura et al. demonstrated a serum pentosidine level more marked progressively in microalbuminuria and advanced stages of nephropathy [88]. Bruce A et al. found higher excretion in urine among patients with microalbuminuria and early decline of GFR [89]. Diabetic patients with a high level of pentosidine were found to be an independent predictor of diabetic retinopathy, cardiovascular disease, and all-cause mortality [90, 91]. Lines to this evidence, measurement of pentosidine level in urine and serum may provide the basis for identifying patients at risk of early GFR decline and could be a promising biomarker for diabetic microvascular and macrovascular complications.

### Uric acid

Uric acid is produced by purine metabolism and has been shown to play an independent function in predicting DKD progression and many clinical studies have been focused targeting its level in the prognosis of DKD. Bartakova et al. found initial hyperuricemia is a strong determinant of DKD progression [92]. Zoppini et al. analyzed that the cumulative incidence of CKD with GFR decline among T2DM was significantly higher in those who had hyperuricemia, considered as an independent risk factor in disease progression and as a strong predictor of GFR decline [93]; furthermore, T1DM with higher serum uric acid levels were developed persistent macroalbuminuria [94]. These evidences suggest that serum uric acid could be an independent predictor of later development of macroalbuminuria in type 1 and type 2 diabetic patients.

## Biomarkers of inflammation

Recent researchers have reported the potential role of local and systemic inflammatory pathways in the progression of DKD with chronic inflammation and subsequent extracellular matrix expansion [95].

### Tumor necrotic factor-alpha (TNF-α)

TNF-α expresses in glomerular and tubular cells in all stages of diabetes, mainly monocyte-produced cytokines, and predisposes in all the stages of the pathogenesis of DKD progression by inducting and infiltrating inflammatory cells to the kidney and activation of apoptosis system. Thereby elevated level of TNF-α has been noted with hypertrophy, hyperfiltration, and alterations of intra-glomerular blood flow, resulting in reduced renal function [95].

A meta-analysis by Qiao et al. reported T1DM patients have significantly increased TNF-α as compared to healthy controls [96]. Furthermore, Navarro JF et al. documented that serum TNF-α is elevated with advanced renal dysfunction and correlates with urinary protein excretion, suggesting that this cytokine has an intensive role in the onset of proteinuria in these patients [97]. While Stangou et al. reported a significant positive correlation of urinary TNF-α, but not serum TNF-α with the severity of microalbuminuria in T2DM [98]. An experimental animal, study corroborated the key role of TNF-α in mediating the pathogenesis of diabetic peripheral neuropathy [99]. Elevated TNF-α is also associated with microvascular and macrovascular complications in diabetic patients [100, 101] and in the prediction of diabetic retinopathy in T2DM with an AUC of 0.84 [102]. The above studies suggest that serum and urine TNF-α could be a potential biomarker to predict the degree of microalbuminuria in T1DM and T2DM.

### Tumor necrotic factor-alpha receptors

TNF-α receptors are type1 transmembrane proteins with cysteine-rich motifs seen in glomerular and tubular cells. These are of two types, TNF-α receptor 1 (55 kDa) and TNF -α receptor 2 (75 kDa). TNF-α binds to these respective receptors and induces inflammatory pathways and apoptosis [103].

Current studies have appreciated the contribution of TNF-α receptors on the magnitude of DKD development through the TNF*α*–TNFR2 inflammatory pathways [103]. Sharad et al. found a strong correlation of serum TNF-α receptors with microalbuminuria among T1DM, suggesting the crucial role in disease progression [104]. These findings have been supported by evidence of a marked stepwise increase from normo-micro-macroalbuminuria in T2DM [105]. Multiple studies have reported that TNFRs are associated independently with declined renal function and ESRD [106]. Furthermore, it has been described that serum TNF receptors are predictors of diabetic retinopathy in T1DM [107].

Therefore, circulating TNF-α receptors are associated with the progression of DKD and could be a predictor of microalbuminuria and advanced renal impairment.

### Monocyte chemoattractant protein-1 (MCP-1)

MCP-1 is a pro-inflammatory cytokine produced by mononuclear leukocytes, cortical tubular epithelial cells, and podocytes that has been linked to renal inflammation, glomerular injury, tubular atrophy, and fibrosis via nuclear factor-kappa B [108]. Renal expression of MCP-1 was also correlated with the quantity of infiltrated macrophages, interstitial lesions, and the degree of albuminuria [109]. Fufaa et al. demonstrated a substantial correlation of urine MCP-1 (uMCP-1) levels with cortical interstitial expansion and disease progression in T1DM who had normoalbuminuria [110]. When comparing DKD patients to healthy controls, Wada [111] and Banba [112] discovered elevated urinary excretion of MCP-1 in DKD patients. Shoukry et al., have observed increased uMCP-1 in T2DM progressively from normo-micro-macroalbuminuria and its early diagnostic utility with 96% sensitivity and 84% specificity [113]. Early progressive GFR decline has a positive correlation with high uMCP-1 [114] also associated with young-onset of T2DM with diabetic retinopathy [115]. These observations suggest that MCP-1 could be a promising inflammatory marker in diagnosing early progressive renal decline and diabetic microvascular complications.

### Transforming growth factor-beta (TGF- β)

TGF-β activates fibrogenesis and thereby progression of DKD by the increased extracellular matrix deposition and glomerular mesangial hypertrophy [116].

Flores et al. have shown raised urinary and plasma TGF- β in clinical onset of T1DM [117]. Similarly, patients with T2DM who had microalbuminuria had been reported elevated serum and urinary TGF-β [118]. On the other hand, Rivarola et al. found increased urinary TGF-β in type 2 diabetic patients with persistent proteinuria (> 500 mg/24 h) than the microalbuminuric group [119]. Supporting this, a study conducted among T2DM resulted in increased serum and urine TGF-β, being more pronounced in macroalbuminuria compared to microalbuminuria and normoalbuminuria group [120], suggesting that this biomarker could be a good candidate in predicting macroalbuminuria in type 2 DM.

### Connective tissue growth factor (CTGF)

CTGF is a secretory protein in renal cells induced by hyperglycemia. It stimulates extracellular matrix synthesis, cell migration, and interstitial matrix deposition by the epithelial-to-mesenchymal transition in diabetic patients [121].

T1DM patients showed increased urinary CTGF with the severity of renal function deterioration in terms of albumin excretion and GFR decline [122] and could be a predictor for ESRD with an AUC under ROC of 0.72 [123]. Its expression has also been reported in diabetic retinopathy [124]. CTGF could be an independent predictor of ESRD and mortality in DKD and it is also a predictor of diabetic retinopathy and looks promising.

### Interleukins-6 (IL-6)

It is a major immunoregulatory cytokine in mesangial expansion. Sangoi et al. observed higher serum IL-6 even before the onset of albuminuria [125]. Multiple studies support these findings with evidence that serum and urinary IL-6 were increasing with disease progression in T1DM [126] and T2DM [127] and have a pronounced association with macrovascular complications [101]. Thus, it could be a marker of the onset of microalbuminuria and early progressive renal decline, and it strongly predicts the macrovascular complications of diabetes.

The classification based on pathogenesis and utilization of these biomarkers can be the future in predicting the early onset of microalbuminuria and progressive renal function decline in both T1DM and T2DM. These biomarkers are relevant to not only predicting the progression of DKD but also diabetic microvascular and macrovascular complications, as shown in Table 2. Few of the biomarkers have been studied in the type 2 diabetic population on their diagnostic utility for DKD and established cut-off value with the area under the ROC curve from 0.744 to 0.99, which are listed down in Table 3

**Table 2:** Overview of biomarkers for prediction of onset, progression, and diabetic complications

Type of biomarkers	Name of biomarkers	Utility in DKD prediction	Prediction of retinopathy	Prediction of cardiovascular risk
Glomerular marker	Fibronectin	Progression (T1DM, T2DM) [22, 23]	Yes [22]	Yes [24]
	Type IV collagen	Onset (T1DM, T2DM) [16] Progression (T2DM) [17]	Yes [20]	n/a
	Serum laminin	Onset (T2DM) [26] Progression (T2DM) [27]	Yes [28]	n/a
	Serum cystatin C	Onset (T1DM, T2DM) [30, 31] Progression (T1DM, T2DM) [32]	Yes [33]	Yes [34]
	GAG	Onset and progression (T1DM, T2DM) [38]	Yes [39]	n/a
	Transferrin	Progression (T2DM) [53]	Yes [54]	Yes [55]
Tubular markers	NGAL	Onset and progression (T2DM) [57]	n/a	n/a
	RBP-4	Onset and progression (T1DM, T2DM) [74, 78]	Yes [75]	Yes [72]
	KIM-1	Onset (T2DM) [69] Progression (T1DM) [90]	n/a	n/a
	L-FABP	Progression (T2DM) [99]	n/a	Yes [79]
Oxidative marker	8-OxodG	Progression (T2DM) [104]	Yes [84]	Yes [86]
	Pentosidine	Progression (T1DM, T2DM) [110, 111]	Yes [128]	Yes [91]
Inflammation	MCP-1	Progression (T2DM) [113]	Yes [115]	n/a
	TNF-**α**	Progression (T2DM) [98]	Yes [100]	Yes [101]
	TNF-**α** Receptors	Progression (T1DM, T2DM) [104, 129]	Yes [107]	n/a
	CTGF	Predictor of ESRD (T1DM) [123]	Yes [124]	n/a
	IL-6	Onset and progression (T2DM) [127]	n/a	Yes [101]

*GAG* glycosaminoglycans, *RBP-4* retinol-binding protein-4, *8-OxodG* 8-oxo-7,8-dihydro-2-deoxyguanosine, *MCP-1* monocyte chemoattractant protein- 1, *TNF-****α*** tumor necrotic factor- **α**, *TNF- ****α**** receptors* tumor necrotic factor- α receptors; *n/a* not application

**Table 3:** Overview of biomarkers for the diagnostic utility of DKD in type 2 diabetic population

Study	Biomarkers	Patients	Cut-off value	AUC	Sensitivity (%)	Specificity (%)	Method
Rashid et al. 2021 [27]	Serum laminin	96	135.7 pg/ml	0.913	85	80	ELISA
Kaul et al. 2018 [57]	Urine NGAL	198	21.31 mg/dl	0.996	95	100	ELISA
Qamar et al. 2018 [31]	Serum cystatin C	119	1.26 mg/L	0.914	88.2	84.8	Turbidimetric
Xian et al. 2017 [61]	Urine cystatin C	146	0.525 ng/ml	0.803	0.76	0.80	Turbidimetric
Ali et al. 2018 [69]	uKIM-1	89	88.5 ng/l	–	91.6	84	ELISA
Abbasi et al. 2020 [62]	uRBP-4	133	46.1 ng/mg	0.744	84.6	62.5	ELISA
Shoukry et al. 2015 [113]	uMCP-1	100	110 pg/mg	0.98	92	100	ELISA

*NGAL* neutrophil gelatinase-associated lipocalin, *KIM-1* kidney injury molecule-1, *RBP4* retinol-binding protein, *8-OxodG* 8-oxo-7,8-dihydro-2-deoxyguanosine, *MCP-1* monocyte chemoattractant protein- 1, *TNF-****α*** tumor necrotic factor-**α**

## Emerging biomarkers

### Microparticles/microvesicles (MPs)

These are 0.1- to 1.0-μm, spherical in structure and formed by the extravascular budding of the plasma membrane by the stimulation to external factors such as inflammation and apoptosis and thereby reorganization of the cytoskeleton [129]. Under hyperglycaemic injury, renal cells are activated to release MPs into plasma and urine before the onset of DKD [130]. Therefore, these have emerged as biomarkers in DKD.

A review by Sheyu Li et al. reported a higher level of circulating MPs reported with T2DM and as independent predictors for microvascular complications of diabetes [131]. MPs can be easily isolated from body fluids via non-invasive methods. These properties facilitate the use as a potential non-invasive biomarker in the progression of DKD. More large-scale studies are needed for further relevance in this regard.

### Urinary exosomes

Urinary exosomes, 40–100 nm originate as internal vesicles, and that contain protein indicators of renal failure and structural damage. It has turned out to be a potential non-invasive biomarker source. However, exosome isolation was challenging. Using liquid chromatography and mass spectrometry, the scope of existing approaches has enlarged to evaluate more urinary exosome-associated proteins [132].

### microRNA

miRNAs are short non-coding RNAs that influence gene expression through epigenetic and post-transcriptional processes carried by exosome/microvesicle. In recent years, microRNA is reported to be involved in the DKD progression via inflammation, hypertrophy, autophagy, endoplasmic reticulum (ER) stress, oxidative stress, insulin resistance, and podocyte injury. Jia et al. observed a positive correlation of miRNA expression for TGF beta stimulator with albuminuria and reported good diagnostic efficiency [133]. Based on the evidence mentioned above, urine mRNA has prognostic significance as a non-invasive, early indicator of renal impairment.

## Conclusion

The pathophysiology of DKD and its progression is multifactorial. Therefore, assessment of development and progression of DKD cannot be relied solely on albuminuria and creatinine. The identification of novel biomarkers based on pathogenesis of DKD involving various renal structures looks promising. In this review, we have summarized the potential 22 novel biomarkers with respect to the pathogenesis of DKD development. Each biomarker has its role in either identifying DKD early or predicting progression of DKD over and above clinical history and standardized markers like albuminuria and creatinine. Few of them appear to be useful for predicting other micro- and macrovascular complications like retinopathy and cardiovascular disease. This panel of biomarkers now warrants further validation on large-scale longitudinal studies involving type 1 and type 2 diabetes populations before the transition to clinical routine.
